# Supplementary material for: Homophilic wiring principles underpin neuronal network topology in vitro
Source: eLife. 2025 Jul 8;14:e85300. doi: 10.7554/eLife.85300 (PMC12563572; doi:10.7554/eLife.85300)
Supplement: Supplementary file 1. — (a) Overview of the datasets used in the study. (b) A list of all the value Ki,j terms that were included in the generative modeling, as given in the wiring equation. (c) A summary of all optimized parameters and energy values, for each dataset, across generative rules. (d) Statistical comparisons of the sparse rodent culture energy values across generative rules. (e) Statistical comparisons of the sparse rodent culture topological fingerprint dissimilarity across generative rules. (f) Statistical comparisons of the dense rodent culture topological fingerprint dissimilarity across generative rules. (g) Statistical comparisons of the dense rodent culture energy values across generative rules. (h) Statistical comparisons of human monolayer neuron and cerebral organoid culture energy values across generative rules. (i) Overview of used antibodies. (j) Confidence intervals of model energies, generated by subsampling from the dense rodent networks. [file elife-85300-supp1.docx]

SUPPLEMENTARY FILE 1

Homophilic wiring principles underpin
neuronal network topology *in vitro*

Danyal Akarca^1,2,3^*, Alexander W. E. Dunn^4,5^*, Philipp J. Hornauer^6^, Silvia Ronchi^6^, Michele Fiscella^6^, Congwei Wang^7^, Marco Terrigno^7^, Ravi Jagasia^7^, Petra E. Vértes^8^, Susanna B. Mierau^4,9,10^, Ole Paulsen^4^, Stephen J. Eglen^5^, Andreas Hierlemann^6^, Duncan E. Astle^1,11^**^†^**, Manuel Schröter^6^**^†^**

1. MRC Cognition and Brain Sciences Unit, University of Cambridge, Cambridge, UK
2. Department of Electrical and Electronic Engineering, Imperial College London, London, UK
3. I-X, Imperial College London, London, UK
4. Department of Physiology Development and Neuroscience, University of Cambridge, Cambridge, UK
5. Department of Applied Mathematics and Theoretical Physics, University of Cambridge, Cambridge, UK
6. Department of Biosystems Science and Engineering in Basel, ETH Zurich, Switzerland
7. NRD, Roche Innovation Center Basel, F. Hoffmann-La Roche Ltd., Basel, Switzerland
8. Department of Psychiatry, University of Cambridge, Cambridge, UK
9. Division of Cognitive and Behavioral Neurology, Brigham & Women’s Hospital, Boston, USA
10. Harvard Medical School, Boston, USA
11. Department of Psychiatry, University of Cambridge, Cambridge, UK

Corresponding authors:

Dr Danyal Akarca

Email: [danyal.akarca@mrc-cbu.cam.ac.uk](mailto:danyal.akarca@mrc-cbu.cam.ac.uk)

Dr Manuel Schröter

Email: [manuel.schroeter@bsse.ethz.ch](mailto:manuel.schroeter@bsse.ethz.ch)

*Co-lead first authors

**†**Co-lead senior authors

# CONTENTS

## Supplementary files

**Supplementary file 1a.** Overview of the datasets used in the study.

**Supplementary file 1b.** A list of all the value *K_i,j_* terms that were included in the generative modeling, as given in the wiring equation.

**Supplementary file 1c.** A summary of all optimized parameters and energy values, for each dataset, across generative rules.

**Supplementary file 1d.** Statistical comparisons of the sparse rodent culture energy values across generative rules.

**Supplementary file 1e.** Statistical comparisons of the sparse rodent culture topological fingerprint dissimilarity across generative rules.

**Supplementary file 1f.** Statistical comparisons of the dense rodent culture topological fingerprint dissimilarity across generative rules.

**Supplementary file 1g.** Statistical comparisons of the dense rodent culture energy values across generative rules.

**Supplementary file 1h.** Statistical comparisons of human monolayer neuron and cerebral organoid culture energy values across generative rules.

**Supplementary file 1i**. Overview of used antibodies.

**Supplementary file 1j.** Confidence intervals of model energies, generated by subsampling from the dense rodent networks.

| **Cell line** | **Recording time points** | **Cells plated** | **Type of plating** | **Cells density**  **(cells/mm^2^)** | **Type of MEA** | **Samples** |
| --- | --- | --- | --- | --- | --- | --- |
| **Primary rodent cortex (sparse)** | DIV 7, 10, 12, 14 | 50,000 | Whole-array plating | ~1,000 | MaxTwo  (6-well plates) | 6 |
| **Primary rodent cortex (dense)** | DIV 14/15, 28 | 100,000 | Whole-array plating | ~2,000 | MaxTwo (6-well plates) | 12 |
| **Human motor neurons** | DIV 28 | 100,000 | Dot plating | ~10,000 | MaxOne (single wells) | 7 |
| **Human glutamatergic neurons** | DIV 28 | 100,000 | Dot plating | ~10,000 | MaxOne (single wells) | 8 |
| **Human dopaminergic neurons** | DIV 28 | 100,000 | Dot plating | ~10,000 | MaxOne (single wells) | 6 |
| **Human cerebral organoids** | 120 days | - | - | - | MaxTwo (6-well plates) | 6 |

***Supplementary file 1.***

**Overview of the datasets used in the study.**

The plating density here reflects the number of cells plated, rather than the graph density of the connectivity of the functional networks that are subsequently inferred following developmental time.

**
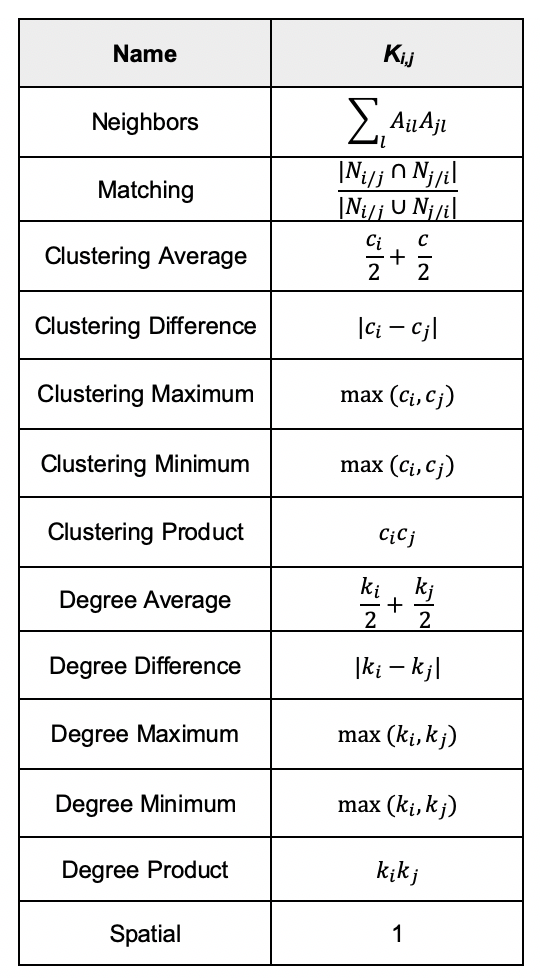
**

***Supplementary file 2.***

**List of all value *K_i,j_* terms that were included in the generative modeling, as given in the wiring equation.**

*A* is the binary adjacency matrix, c is the local clustering coefficient, *k* is the node degree and *N_i_*_/_*_j_* represents the neighbors of node *i*, excluding node *j*. Note that the spatial model enforces *K_i,j_* =1, which means that the value term has no effect on the generative process.

******

***Supplementary file 3.***

**A summary of all optimized parameters and energy values, for each dataset, across generative rules.**

Each dataset is reported alongside the featured figure (s) they are presented in. This table provides a summary of the model performances, but all individual model data is available within our data repository.

**
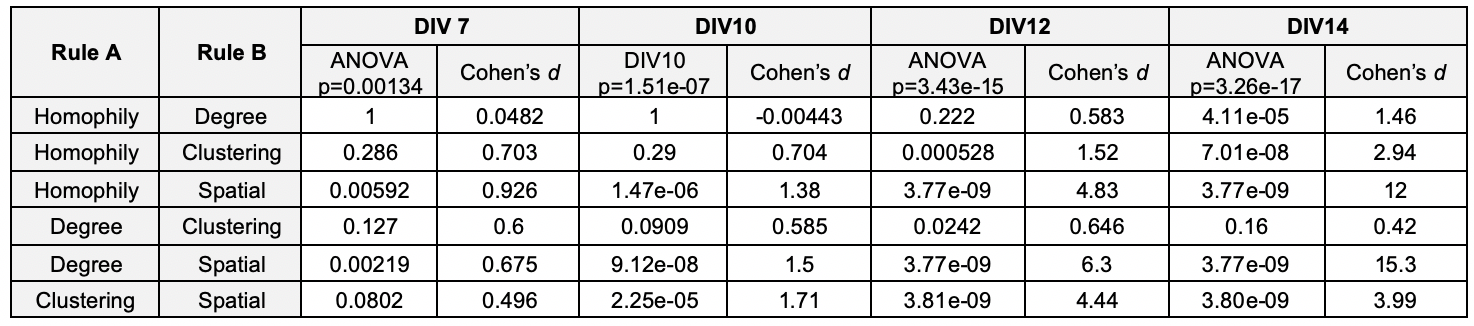
**

***Supplementary file 4.***

**Statistical comparisons of the sparse rodent culture energy values across generative rules.**

For each test, we quote the ANOVA p-value across the generative rules and the corresponding Cohen’s *d* if the ANOVA was significant at p<0.05. A positive Cohen’s *d* reflects that Rule A has a smaller energy than Rule B, reflecting a better fit. Generative rules have been binned across the generative model class.


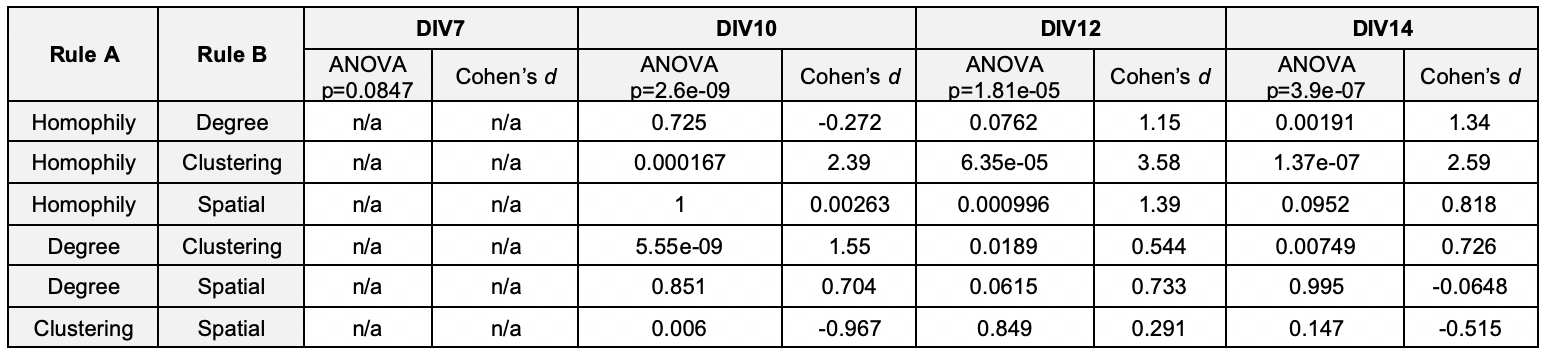


***Supplementary file 5.***

**Statistical comparisons of sparse rodent culture topological fingerprint dissimilarity across generative rules.**

For each test, we quote the ANOVA p-value across the generative rules and the corresponding Cohen’s *d* if the ANOVA was significant at p<0.05. A positive Cohen’s *d* reflects that Rule A has a smaller energy than Rule B, reflecting a better fit. Generative rules have been binned across the generative model class.


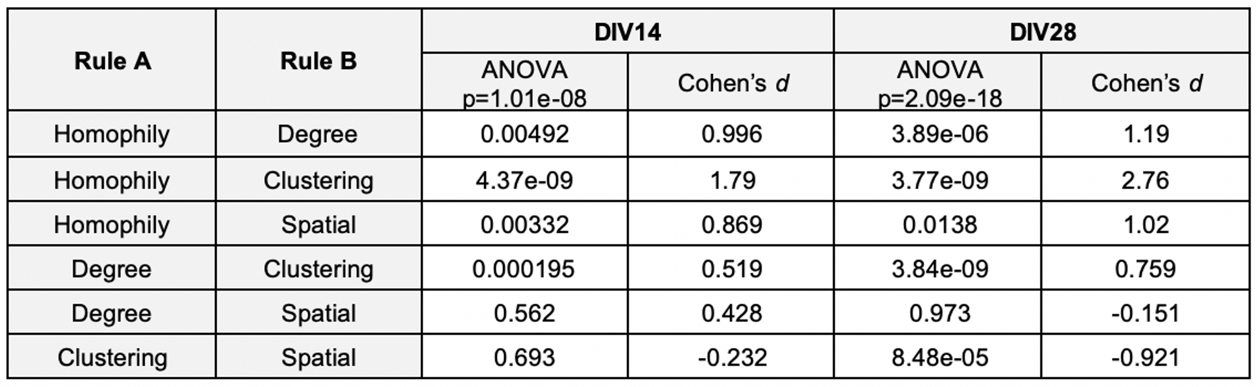


***Supplementary file 6.***

**Statistical comparisons of dense rodent culture topological fingerprint dissimilarity across generative rules.**

For each test, we quote the ANOVA p-value across the generative rules and the corresponding Cohen’s *d* if the ANOVA was significant at p<0.05. A positive Cohen’s *d* reflects that Rule A has a smaller energy than Rule B, reflecting a better fit. Generative rules have been binned across the generative model class.

**
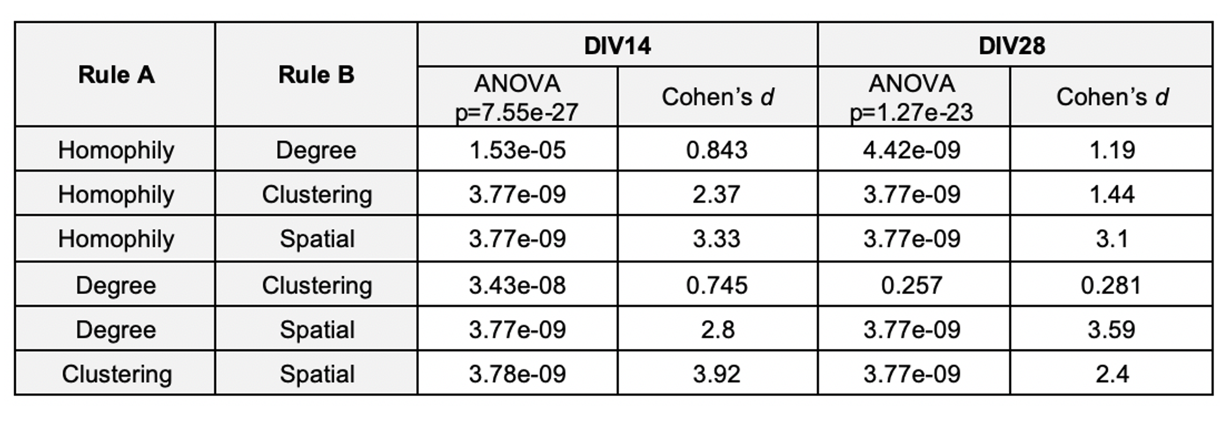
**

***Supplementary file 7.***

**Statistical comparisons of dense rodent culture energy across generative rules.**

For each test, we quote the ANOVA p-value across the generative rules and the corresponding Cohen’s *d* if the ANOVA was significant at p<0.05. A positive Cohen’s *d* reflects that Rule A has a smaller energy than Rule B, reflecting a better fit. Generative rules have been binned across the generative model class.

**
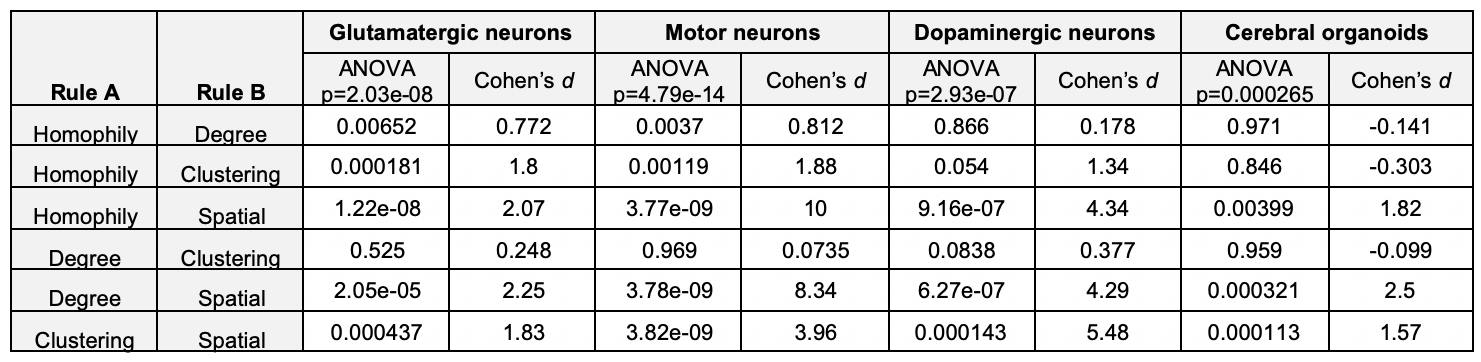
**

***Supplementary file 8.***

**Statistical comparisons of human monolayer neuron and cerebral organoid culture energy values across generative rules.**

For each test, we quote the ANOVA p-value across the generative rules and the corresponding Cohen’s *d* if the ANOVA was significant at p<0.05. A positive Cohen’s *d* reflects that Rule A has a smaller energy than Rule B, reflecting a better fit. Generative rules have been binned across the generative model class.

| **Antibodies (human organoids)** | **Type** | **Dilution** | **Catalog number** |
| --- | --- | --- | --- |
| Tau | Primary | 1:500 | #MN1000, ThermoFisher |
| NeuN | Primary | 1:300 | #M11954-3, Boster Bio, Pleasanton, CA, USA |
| GFAP | Primary | 1:500 | #NB300-141, Novus Biologicals, Englewood, CO, USA |
| goat anti-mouse IgG, Alexa Fluor Plus 488 | Secondary | 1:400 | #A32723, ThermoFisher |
| goat anti-rabbit IgG, Alexa Fluor 568 | Secondary | 1:400 | #A11036, ThermoFisher |
| goat anti-chicken IgY, Alexa Fluor Plus 647 | Secondary | 1:400 | #A32933, ThermoFisher |
| **Antibodies (human iPSC)** | **Type** | **Dilution** | **Catalog number** |
| mouse anti-TH | Primary | 1:500 | #MAB318, Sigma-Aldrich |
| chicken anti-MAP2 | Primary | 1:1000 | #CH22103, Neuromics (Edina, MN, USA) |
| rabbit anti-GFAP | Primary | 1:500 | #Z0334, Agilent (Santa Clara, CA, USA) |
| donkey anti-mouse 488 | Secondary | 1:250 | #A-21202, ThermoFisher |
| goat anti-chicken 647 | Secondary | 1:500 | #A-32933, ThermoFisher |
| donkey anti-rabbit 568 | Secondary | 1:250 | #A-A10042, ThermoFisher |
| **Antibodies (rodent PC)** | **Type** | **Dilution** | **Catalog number** |
| mouse anti-Synaptophysin | Primary | 1:100 | #ab8049, Abcam |
| rabbit anti-NeuN | Primary | 1:300 | #ab177487, Abcam |
| chicken anti-beta III Tubulin | Primary | 1:1000 | #ab41489 Abcam |
| donkey anti-mouse IgG, Alexa Fluor 488 | Secondary | 1:500 | #ab150105, Abcam |
| donkey anti-Chicken IgY (IgG) | Secondary | 1:400 | #703–165-155, Jackson ImmunoResearch, West Grove, USA |
| donkey anti-rabbit IgG, Alexa Fluor 405 | Secondary | 1:1000 | #ab175651, Abcam |

***Supplementary file 9.***

**Primary and secondary antibodies.**

***
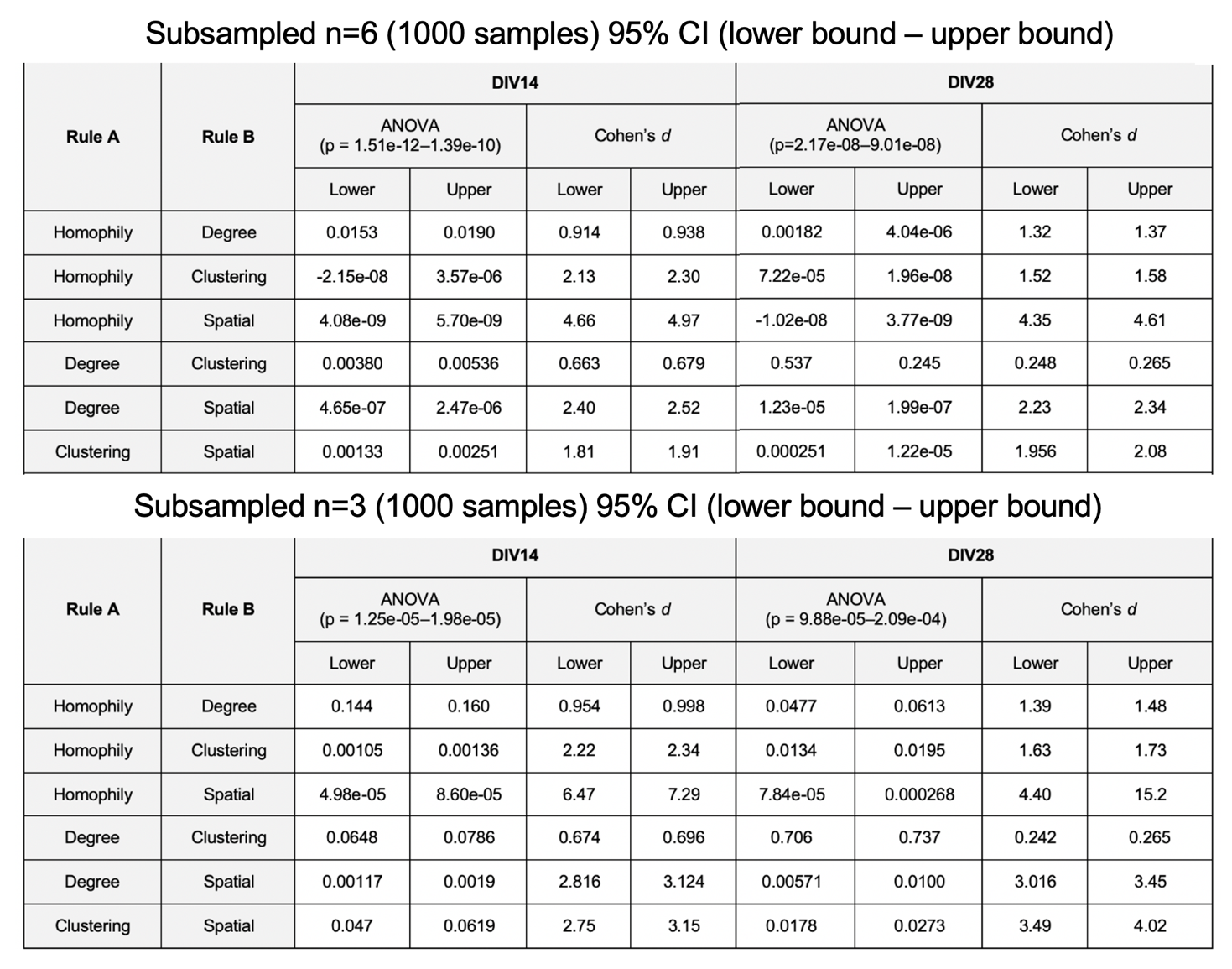
***

***Supplementary file 10.***

**Confidence intervals of model energies, generated by subsampling from the dense rodent networks.**
